# Effect of Rotator Cuff Deficiencies on Muscle Forces and Glenohumeral Contact Force After Anatomic Total Shoulder Arthroplasty Using Musculoskeletal Multibody Dynamics Simulation

**DOI:** 10.3389/fbioe.2021.691450

**Published:** 2021-07-05

**Authors:** Zhenxian Chen, Xunjian Fan, Yongchang Gao, Jing Zhang, Lei Guo, Shibin Chen, Zhongmin Jin

**Affiliations:** ^1^Key Laboratory of Road Construction Technology and Equipment of MOE, Chang’an University, Xi’an, China; ^2^Institute of Mechanical Manufacturing Technology, China Academy of Engineering Physics, Mianyang, China; ^3^State Key Laboratory for Manufacturing Systems Engineering, School of Mechanical Engineering, Xi’an Jiaotong University, Xi’an, China; ^4^Institute of Medical and Biological Engineering, School of Mechanical Engineering, University of Leeds, Leeds, United Kingdom; ^5^Tribology Research Institute, School of Mechanical Engineering, Southwest Jiaotong University, Chengdu, China

**Keywords:** anatomic total shoulder arthroplasty, biomechanics, rotator cuff deficiency, musculoskeletal multibody dynamics, arm abduction

## Abstract

Anatomic total shoulder arthroplasty (ATSA) is widely used to treat the diseases of the glenohumeral (GH) joint. However, the incidence of rotator cuff tears after ATSA increases during follow-up. The effects of rotator cuff deficiencies after ATSA on the biomechanics of the GH joint are to be investigated. In this study, a musculoskeletal multibody dynamics model of ATSA was established using a force-dependent kinematics (FDK) method. The biomechanical effects were predicted during arm abduction under different rotator cuff deficiencies. The deltoid forces were increased under the rotator cuff deficiencies, the maximum deltoid forces were increased by 36% under the subscapularis deficiency and by 53% under the supraspinatus, infraspinatus, subscapularis, and teres minor deficiencies. The maximum GH contact forces were decreased by 11.3% under supraspinatus and infraspinatus deficiencies but increased by 24.8% under subscapularis deficiency. The maximum subscapularis force was decreased by 17% under only infraspinatus tear during arm abduction. The results suggested that the changes in the biomechanics of the GH joint induced by rotator cuff deficiencies after ATSA increase the deltoid muscle energy expenditure and joint instability, which result in postoperative less satisfactory clinical outcomes. The changes in rotator cuff muscle forces deserve more attention for understanding the evolution of rotator cuff tear after ATSA.

## Introduction

Shoulder arthroplasty has become the third most common orthopedic procedure after hip and knee joint arthroplasties (Smith et al., 2015; Simovitch et al., 2017). Two entirely different procedures with different implant designs, anatomic total shoulder arthroplasty (ATSA), and reverse total shoulder arthroplasty (RTSA), are widely used for a variety of joint diseases of the glenohumeral (GH) joint. Compared with the inverted ball-and-socket design of RTSA, the implant of ATSA is designed by emulating the non-conforming anatomy of the shoulder joint. As the best option to salvage shoulders with rotator cuff arthropathy, massive irreparable rotator cuff tears, and tumor resection, etc., RTSA is becoming popular (Merolla et al., 2018) and has a remarkable rise in recent years (Simovitch et al., 2017). ATSA is most commonly adopted in cases of chronic arthritic conditions of the shoulder with intact rotator cuff and produces a well-pleasing mid-to-long-term clinical outcome (Thomas et al., 2018). The function and integrity of the rotator cuff muscles, consisting of the supraspinatus, infraspinatus, subscapularis, and teres minor muscles, play a crucial role in providing dynamic stability to the postoperative GH joint of ATSA (Nam et al., 2012; Alireza et al., 2015). However, the rotator cuff tear is the recognized complication following ATSA in addition to periprosthetic fracture, component loosening, and joint instability (Young et al., 2012; Sheth et al., 2019). A rate of 16.8% was reported at a mean follow-up of 8.6 years for secondary rotator cuff tears after primary ATSA (Young et al., 2012). The rotator cuff dysfunction rate is significantly increasing with the duration of follow-up (Young et al., 2012).

In the clinic, supraspinatus tears are frequently involved in rotator cuff tears with a similar rate as supraspinatus and infraspinatus tears (Hattrup et al., 2006). Teres minor dysfunction affects the external rotation of the humerus and stability of the GH joint (Collin et al., 2015; Kim et al., 2016b). Massive subscapularis tendon tears increase the risk of pseudoparalysis in patients (Collin et al., 2014). Rotator cuff tears are associated with pain, joint instability, and weakness of arm elevation (Hattrup et al., 2006). So postoperative rotator cuff deficiency is bound to affect clinical outcomes and the biomechanics of ATSA. Altered muscle constraints around the GH joint resulting from rotator cuff deficiency associate with the changes of the postoperative joint loading and kinematics. The biomechanical responses of previous joint functional impairments may further aggravate the rotator cuff damage. However, the previous biomechanical studies of ATSA mainly focused on the effects of implant design on bone stress (Razfar et al., 2016), joint force and kinematics, and muscle force (Sins et al., 2015), and the biomechanical benefits of humeral head component anterior-offsetting technique (Kim et al., 2016a). De Wilde et al. (2004) investigated the GH force and deltoid force of ATSA under a non-classified rotator cuff tear. Sins et al. (2016) investigated the effect of subscapularis tear on GH contact patterns of ATSA. But the influences of rotator cuff tears on muscle force and GH contact force are still mainly quantified for RTSA (Ackland et al., 2018). In summary, most clinical studies (Sajadi et al., 2010; Young et al., 2012; Sheth et al., 2019) have discussed the rotator cuff tears after primary ATSA and an incidence rate of 16.8% was reported (Young et al., 2012). However, few studies have been performed to investigate the biomechanics of ATSA with rotator cuff deficiencies.

Although *in vitro* experimental studies can provide valuable information regarding joint loading with (Parsons et al., 2002; Dyrna et al., 2018) and without rotator cuff tears (Ackland et al., 2019), the experimental cost is not conductive to parameter research. Musculoskeletal multibody dynamics modeling provides a non-invasive strong platform for understanding *in vivo* biomechanics of the shoulder and the effects of joint replacement on function. Most of the previous 3D anatomic shoulder musculoskeletal models address GH loading in the light of rotator cuff tears (Holscher et al., 2016; Klemt et al., 2018; Vidt et al., 2018), and these outcomes can be transferable to ATSA. Lemieux et al. (2013) and De Wilde et al. (2004) predicted the GH force and deltoid force of ATSA using musculoskeletal models. Moreover, Sins et al. (2015) introduced successfully the adapted musculoskeletal model of the non-conforming shoulder joint for quantifying joint force and kinematics using the force-dependent kinematics (FDK) method. The predictive ability of the FDK method also was evaluated in previous musculoskeletal simulations of total knee replacement (Chen et al., 2014, 2016). The above studies would pave a way for investigating the biomechanics of ATSA in GH contact force, joint motion, and muscle force under a musculoskeletal dynamics environment.

This study aimed to establish a musculoskeletal multibody dynamic model of ATSA, and further quantify the effects of different rotator cuff deficiencies on muscle forces and GH contact force during arm abduction.

## Materials and Methods

**FIGURE 1:**
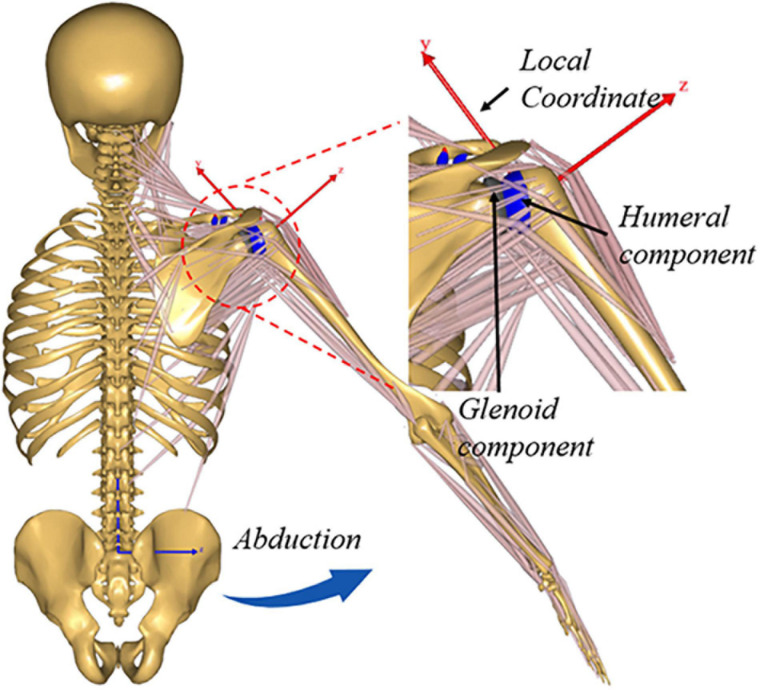
Illustration showing upper extremity musculoskeletal multibody dynamics models of Anatomic total shoulder arthroplasty (ATSA).

A generic upper extremity musculoskeletal model was extracted from Anybody Managed Model Repository (AMMR, V1.6.2) to establish the musculoskeletal multibody dynamics model of ATSA in AnyBody Modeling System (AnyBody Technologies, Aalborg, Denmark, V6.0). There were 118 muscle-tendon units and five joints (acromioclavicular joint, sternoclavicular joint, GH joint, elbow joint, and wrist joint) in the musculoskeletal shoulder model. The deltoid muscles included two parts, deltoideus_clavicular and deltoideus_scapular. The acromioclavicular joint and the sternoclavicular joint were modeled as ball-and-socket joints only allowing three rotational degrees of freedom (DoFs), the elbow joint and wrist joint were modeled as revolute joints only allowing flexion-extension rotation. The DoFs of the GH joint in directions of anterior-posterior, superior-inferior, and medial-lateral translations were released using the FDK method (Andersen et al., 2011; Chen et al., 2016). A linear spring element with a stiffness (Debski et al., 1999) of 1.74 × 10^4^ N/m was established to simulate the passive restriction of the joint capsule and ligaments around the GH joint. A 20 N tolerance value for the FDK residual forces was adopted. A quasi-static equilibrium at every simulation step was found for the FDK solver and iteratively searched until force residuals fell below the tolerance value. A 48 mm diameter humeral head against a polyethylene insert with an 8 mm mismatch was established using CAD software (SolidWorks, Dassault Systems) based on the BIOMODULAR implant (Biomet, Germany). A local reference frame was defined at the joint center of the GH joint as indicated in Figure 1. The implant geometries were incorporated into the musculoskeletal shoulder model by simulating the standard surgical procedure of ATSA, the geometries were implanted in the STL format according to the component position to the joint center in the local reference frame (Figure 1). A deformable contact model was defined between the components of the GH joint according to a penalty-based joint contact algorithm proposed by Anybody. The contact force was calculated using a linear force-penetration volume law with a material parameter known as contact pressure module *P*_*V*_ in N/m^3^ in the default FDK computational framework of AnyBody (Chen et al., 2014). The contact force between contact surfaces was computed as the sum of all vertex contact forces of the triangle mesh. The vertex contact force *F*_*i*_ was calculated based on a linear volume *V*_*i*_ approximated using the penetration depth *d*_*i*_ [as shown in Equation (1)].

(1) $$F_{i}=P_{V}{\;}^{*}\;V_{i}$$

(2) $$P_{V}=\frac{F_{i}}{V_{i}}=\frac{p_{i}\,A_{i}}{A_{i}\,d_{i}}=\frac{\left(1-v\right)}{\left(1+v\right)\,\left(1-2\,v\right)\,h}\,\frac{2\,p_{o}}{\varepsilon_{o}\,\left[1+n\,{\left(\frac{p_{i}}{p_{o}}\right)}^{n-1}\right]}$$

According to the elastic foundation theory (Bei and Fregly, 2004), geometric size and non-linear material property of polyethylene components, a contact pressure module of 2.74e11 N/m^3^ were calculated and used in this study [as shown in Equation (2)]. Where *v* and *h* are Poisson’s ratio and the thickness of polyethylene insert, *A*_*i*_ and *p*_*i*_ are the contact area and contact pressure of the opponent triangle for the *i*th vertex. Non-linear polyethylene material parameters of ε_*o*_ = 0.0597, *p*_*o*_ = 18.4 MPa, and *n* = 3 derived in a previous experimental study (Fregly et al., 2003) were used here. More details about FDK modeling of implants can be found in previous studies (Chen et al., 2014, 2016).

Arm abduction was simulated from 0° to 90° based on a driver function defined using a Fourier expansion (Sins et al., 2015) utilizing the established musculoskeletal shoulder model of ATSA. The driver function was of the form as shown in Equation (3) (Sins et al., 2015). *Pos* was the position of the arm relative to the thorax. *A*_*j*_ and *B*_*j*_ were the Fourier coefficients, *ω_*j*_* was the frequency. The increment, corresponded to the arm abduction angle at each step, was calculated using Equation (3). It took 90 s to perform the whole arm abduction motion. The DoFs of thorax, head, and pelvis segments were constrained. The scapula was restrained by simulating a permanent contact between the thorax and the angulus inferior landmark of the scapula (Sins et al., 2015).

(3) $$P\,o\,s=\sum \left[A_{j}\,\cos\left(\omega_{j}\,t+B_{j}\right)\right]$$

The muscle force was predicted by solving a muscle recruitment problem (Damsgaard et al., 2006), and muscle recruitment was the process of determining which set of muscle forces will balance a given external load (Chen et al., 2016). The isometric muscle strength of each muscle in the upper extremity musculoskeletal model was calculated by multiplying the physiological cross-sectional area by a factor of 27 N/cm^2^ for all muscles (Chen et al., 2016). During arm abduction simulation, the contact force and joint translation of the GH joint, and muscle forces, were calculated simultaneously using the quadratic polynomial muscle recruitment criterion. Before the musculoskeletal shoulder model was used to quantify the biomechanics of the GH joint after ATSA under rotator cuff deficiencies, the sensitivity analyses were performed for the key modeling parameters and presented in Supplementary Materials. The maximum changes of the GH joint forces were 9.3, 13.3, 7.6, and 11%, respectively, for parameter variations in the pressure module, muscle recruitment criterion, scaling law, and analysis step. The calculated pressure module, quadratic polynomial muscle recruitment criterion, length-mass-fat scaling law, and default analysis step were adopted ultimately.

The public OrthoLoad experimental data (Bergmann et al., 2011)^1^, which included the measured *in vivo* GH joint forces of six patients during arm abduction using instrumented BIOMODULAR implant, were used to indirectly evaluate the predictive ability of musculoskeletal modeling method of ATSA. The range formed by the measured GH forces of six patients was used to compare with the predicted GH forces. Then rotator cuff intact and five situations of rotator cuff deficiencies were considered to investigate the biomechanics of ATSA. Five situations of rotator cuff deficiencies included: Q1: infraspinatus deficiency; Q2: supraspinatus deficiency; Q3: supraspinatus and infraspinatus deficiencies; Q4: subscapularis deficiency; Q5: supraspinatus, infraspinatus, subscapularis, and teres minor deficiencies. Under each situation, the corresponding muscle activation was lost. The muscle forces and GH contact forces were predicted under different rotator cuff situations during arm abduction.

## Results

**FIGURE 2:**
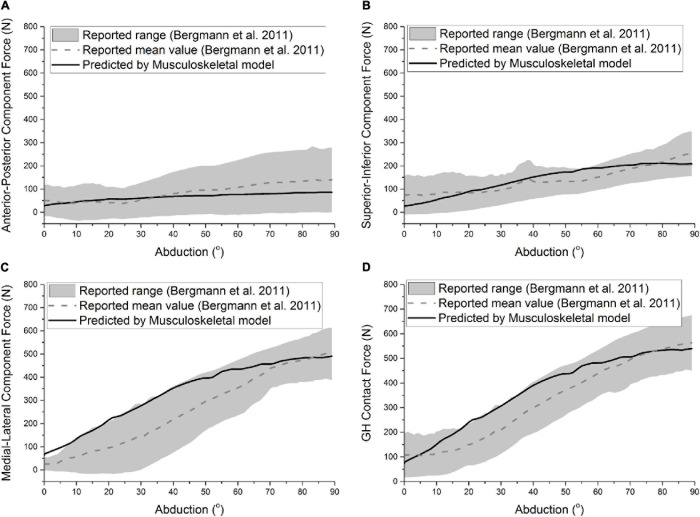
Predicted anterior-posterior component force **(A)**, superior-inferior component force **(B)**, medial-lateral component force **(C)**, and GH contact force **(D)** of ATSA was compared with the results of Bergmann et al. (2011) during the abduction. The gray area and the dashed line represent the range and mean value formed by the measured GH forces of six patients.

The predicted GH contact force and component forces of ATSA by the established musculoskeletal model are compared with the reported range and mean value of the measured *in vivo* GH joint forces of six patients (Bergmann et al., 2011) in Figure 2. Compared with the reported mean value (Bergmann et al., 2011), the computational model was able to predict the anterior-posterior component force [Root-mean-square error *(RMSE)* < 59.2 N], superior-inferior component force (*RMSE* < 90.8 N), medial-lateral component force (*RMSE* < 28.3 N), and GH contact force (*RMSE* < 60.9 N) with reasonable accuracy in trend and amplitude.

**FIGURE 3:**
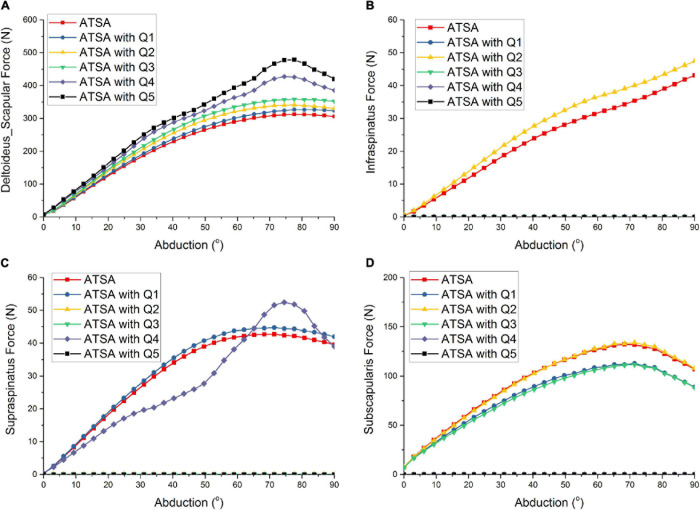
Predicted deltoideus_scapular force **(A)**, infraspinatus force **(B)**, supraspinatus force **(C)**, subscapularis force **(D)** of ATSA under intact rotator cuff and different rotator cuff deficiencies. Q1: infraspinatus deficiency; Q2: supraspinatus deficiency; Q3: supraspinatus and infraspinatus deficiencies; Q4: subscapularis deficiency; Q5: supraspinatus, infraspinatus, subscapularis, and teres minor deficiencies.

Figure 3 shows the predicted deltoideus_scapular forces of ATSA under the five situations of rotator cuff deficiencies. The predicted deltoideus_clavicular forces, not included in Figure 3, were almost zero under the five situations of rotator cuff deficiencies during the arm abduction from 0° to 90°. The predicted deltoideus_scapular forces were all increased under the five situations of rotator cuff deficiencies. The maximum deltoideus_scapular forces of ATSA were increased from 313 N to 479 N. The deltoideus_scapular force was influenced significantly by the subscapularis deficiency (Q4) than infraspinatus deficiency (Q1), supraspinatus deficiency (Q2), and supraspinatus and infraspinatus deficiencies (Q3). The maximum deltoideus_scapular forces were increased by 36% under the subscapularis deficiency (Q4) and by 53% under the supraspinatus, infraspinatus, subscapularis, and teres minor deficiencies (Q5) compared with the intact rotator cuff.

Compared with the intact rotator cuff, the infraspinatus force was increased under only the supraspinatus deficiency (Q2) in Figure 3. There was no influence of the subscapularis deficiency (Q4) on the infraspinatus force. Compared with the intact rotator cuff, the supraspinatus force was increased under only the infraspinatus deficiency (Q1). Although the supraspinatus force was decreased during the arm abduction from 0° to 60° and increased during the arm abduction from 60° to 90° under only the subscapularis deficiency (Q4), the predicted supraspinatus force was the same to the intact rotator cuff at 90° position. Compared with the intact rotator cuff, the maximum subscapularis forces were decreased by 17% under the infraspinatus deficiency (Q1) and supraspinatus and infraspinatus deficiencies (Q3). There was no influence of the supraspinatus deficiency on the subscapularis force. The predicted teres minor forces, not included in Figure 3, were almost zero under the five situations of rotator cuff deficiencies during the arm abduction from 60° to 90°.

**FIGURE 4:**
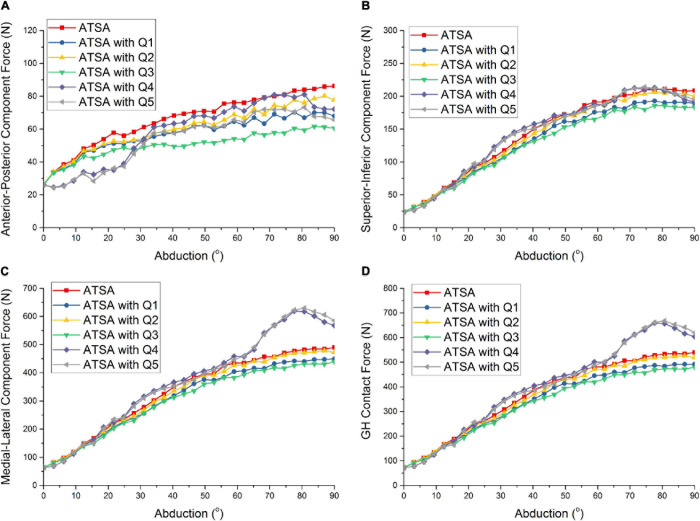
Predicted anterior-posterior component force **(A)**, superior-inferior component force **(B)**, medial-lateral component force **(C)**, and GH contact force **(D)** of ATSA under intact rotator cuff and different rotator cuff deficiencies. Q1: infraspinatus deficiency; Q2: supraspinatus deficiency; Q3: supraspinatus and infraspinatus deficiencies; Q4: subscapularis deficiency; Q5: supraspinatus, infraspinatus, subscapularis, and teres minor deficiencies.

Figure 4 shows the predicted GH contact force and component forces of ATSA under the five situations of rotator cuff deficiencies. Compared with the intact rotator cuff, the predicted GH contact forces of ATSA were decreased by 9.3, 2.6, and 11.3% at the 78°abduction angle in the order from Q1 to Q3. The effect of the infraspinatus deficiency (Q1) on GH contact forces was larger than from the supraspinatus deficiency (Q2). However, the subscapularis deficiency (Q4) decreased the influence of the supraspinatus and infraspinatus deficiencies on the GH contact force and component forces of ATSA. The GH contact forces were increased by 24.8 and 25.2% at the 78° abduction angle under the subscapularis deficiency (Q4) and the supraspinatus, infraspinatus, subscapularis, and teres minor deficiencies (Q5).

## Discussion

A musculoskeletal multibody dynamics model of ATSA was established using the FDK method in this study. The contact mechanics and joint kinematics of the GH joint were considered in the inverse dynamic simulation of the musculoskeletal shoulder model. The contact forces and muscle forces could be calculated simultaneously during arm abduction simulation. The predicted GH contact forces using the musculoskeletal model of ATSA were indirectly evaluated by comparing with the previous reports (Bergmann et al., 2011). Due to the lack of kinematical data of the corresponding patient, the arm abduction was simulated in this study, which may influence the prediction accuracy. Even so, the musculoskeletal model provided a potential approach for understanding the biomechanics of ATSA to improve the implant design of the artificial shoulder joint and study the prosthetic function outcome and failure mechanism.

Rotator cuff deficiency results in an increase of deltoid efficiency during arm abduction (Holscher et al., 2016). The prediction indicated again that the deltoid force was increased along with the rotator cuff deficiency. Especially for a complete rotator cuff deficiency, the deltoid force was increased by more than 53%. The stability of the shoulder joint was reduced, and the changes in the moment arm of muscle led to the increase of deltoid force and a non-monotonic trend (Terrier et al., 2008). These results suggested that the patients with ATSA raise their arm strenuously after rotator cuff tears, which reduces the satisfaction of patients. Although rotator cuff deficiency also results in an increase in deltoid force for RTSA (Terrier et al., 2008), ATSA after rotator cuff tears required more deltoid force to complete the arm abduction. The postoperative rotator cuff tears of ATSA should obtain more surgical attention due to the induced potential clinical pain and strenuous arm elevation.

Although the deltoid force was increased along with the supraspinatus and infraspinatus deficiencies, the GH contact force was decreased. From this, the supraspinatus and infraspinatus are major contributors to the compressive forces of the GH joint. Vidt et al. (2018) had found that smaller peak resultant and compressive forces of the GH joint were generated from the presence of rotator cuff tear. The diminished compressive force was identified for the shoulder with rotator cuff tear which led to less anterior-posterior stability (Lippitt et al., 1993; Vidt et al., 2018). A less anterior-posterior component force was also generated from the presence of rotator cuff tear in this study. However, it is interesting that the GH contact force was increased under the subscapularis deficiency in this study. The subscapularis deficiency resulted in an increase of deltoid force, which might lead to the increase of the GH contact force and a non-monotonic trend. This finding was similar to the previous report by Sins et al. (2016), where the GH reaction force and contact pressure were increased under the subscapularis deficiency compared with the intact rotator cuff. This aspect could be detrimental to the polyethylene insert survival. So, it is very serious for ATSA due to a higher joint force and less stability resulted from the supraspinatus, infraspinatus, and subscapularis deficiencies.

The infraspinatus deficiency and supraspinatus deficiency influenced each other in this study. The mechanical interaction between infraspinatus and supraspinatus may be the main cause of deterioration of infraspinatus and supraspinatus tears. There was no influence of the subscapularis deficiency on the infraspinatus force. However, the subscapularis force was decreased under the infraspinatus deficiency compared with the intact rotator cuff. While there was no influence of the supraspinatus deficiency on the subscapularis force. But the subscapularis deficiency influenced the amplitude fluctuations of the supraspinatus force. From this, the infraspinatus tear would increase the supraspinatus force and decreased the subscapularis force. The decreased subscapularis force further induced the amplitude fluctuations of the supraspinatus force. Therefore, the effect of the infraspinatus tear on the supraspinatus force was compounded. The changes in rotator cuff muscle forces deserve more attention for understanding the evolution of rotator cuff tear in the clinic after ATSA.

Several limitations are ought to be discussed. First, only five situations of rotator cuff deficiencies were considered to investigate the biomechanics of ATSA, the other combined rotator cuff tears and reconstructions were not considered. Second, the arm abduction motion was defined by a simple driver without considering other motion inputs. The motion capture data of the patient should be used to obtain a realistic joint translation. Only abduction motion was simulated in this study, and other more meaningful activities of daily living were not considered. Bergmann et al. (2011) had pointed out the possible difference between using activities of daily living and standardized activity. Third, the effects of the design features of the artificial shoulder joint were not considered in the current study, only one single subject and prosthesis geometry were included. Increasing glenosphere diameter significantly increased deltoid muscle force and joint force, and lateralization increased abduction range of motion (Langohr et al., 2015). The effect of joint diameter on the range of movement can be supported by the similar works of Putame et al. (2019). Fourth, the passive restriction of the joint capsule and ligaments around the GH joint was simulated by a linear spring element, which is the reason for the difference in muscle force curves and GH contact force curves at 0° abduction. Fifth, the effect of rotator cuff tears on joint translation and the center of pressure position of the GH joint should be investigated. Because the loss of rotator cuff function may lead to subluxation, due to the floated rotation center of the humeral component, and subsequently may result in shoulder functional disability. Thus, the shoulder dislocation in the case of ATSA with rotator cuff deficiency should be evaluated. All the above limitations should be considered in the future study. Despite these limitations existed in the current study, the prediction improved the understanding of rotator cuff deficiency after ATSA, and the musculoskeletal model of ATSA provides a strong platform for implant design, preoperative planning, and function evaluation.

## Conclusion

The deltoid forces were increased under the rotator cuff deficiencies, which may induce potential clinical pain and strenuous arm elevation. The GH contact force was decreased under supraspinatus and infraspinatus deficiencies but increased under subscapularis deficiency. Infraspinatus tear would increase the supraspinatus force and decrease the subscapularis force. The decreased subscapularis force further induced the amplitude fluctuations of the supraspinatus force. So, the effect of the infraspinatus tear on the supraspinatus was compounded. The changes in rotator cuff muscle forces deserve more attention for understanding the evolution of rotator cuff tear in the clinic after ATSA.

## Data Availability Statement

The original contributions presented in the study are included in the article/Supplementary Material, further inquiries can be directed to the corresponding author.

## Author Contributions

ZC, XF, YG, JZ, and ZJ conceived and designed the study. ZC and XF performed the simulation and prepared the manuscript. LG, SC, and ZJ reviewed and edited the manuscript. All authors read and approved the manuscript.

## Conflict of Interest

The authors declare that the research was conducted in the absence of any commercial or financial relationships that could be construed as a potential conflict of interest.

## Footnotes

1 https://orthoload.com/database/
