# Supplementary material for: Effect of Rotator Cuff Deficiencies on Muscle Forces and Glenohumeral Contact Force After Anatomic Total Shoulder Arthroplasty Using Musculoskeletal Multibody Dynamics Simulation
Source: Front Bioeng Biotechnol. 2021 Jul 5;9:691450. doi: 10.3389/fbioe.2021.691450 (PMC8287529; doi:10.3389/fbioe.2021.691450)
Supplement: Supplementary file 1 [file Table_1.DOCX]

S-table 1. Sensitivity of glenohumeral joint forces to changes in analysis step, scaling law, muscle recruitment criterion, and material parameter *PressureModule* for MSK model of anatomic total shoulder arthroplasty. *The nominal value for investing the effect of model parameters on joint contact forces by the RMS errors (N) and *ρ* coefficients

| **Parameters** | **Total contact force** | | **Force in x-direction** | | **Force in y-direction** | | **Force in z-direction** | |
| --- | --- | --- | --- | --- | --- | --- | --- | --- |
|  | **RMSE** | ***ρ*** | **RMSE** | ***ρ*** | **RMSE** | ***ρ*** | **RMSE** | ***ρ*** |
| **Anaylysis step**  Default value *  Default value × 2  Default value × 0.5 | 46.50  59.14 | 1  1 | 39.16  17.90 | 0.99  0.99 | 35.99  27.99 | 0.99  1 | 37.69  29.45 | 0.99  1 |
| **Scaling law**  Length  Length-Mass  Length-Mass-Fat * | 40.85  12.21 | 1  1 | 23.78  19.27 | 1  0.99 | 15.66  6.78 | 0.99  0.99 | 13.26  15.48 | 1  1 |
| **recruitment criterion**  MinMax  Cubic polynomial  Quadratic polynomial * | 72.08  57.02 | 1  1 | 54.64  48.29 | 0.99  0.99 | 17.98  15.75 | 0.97  0.97 | 18.18  16.94 | 1  1 |
| ***PressureModule***  Max 4.03e^11^N/m^3^  Min 2.33e^11^N/m^3^  Average 2.74e^11^N/m^3^* | 41.39  50.47 | 0.99  0.99 | 26.30  37.89 | 1  1 | 17.13  19.32 | 0.99  0.98 | 14.03  15.27 | 0.99  0.99 |
